# Communication Training: Significance and Effects of a Preliminary Psychological Intervention upon an Audit Team

**DOI:** 10.3390/ijerph20054173

**Published:** 2023-02-25

**Authors:** Davide Cardile, Augusto Ielo, Francesco Corallo, Irene Cappadona, Giangaetano D’Aleo, Maria Cristina De Cola, Placido Bramanti, Rosella Ciurleo

**Affiliations:** IRCCS Centro Neurolesi Bonino-Pulejo, S.S. 113 Via Palermo, C. da Casazza, 98124 Messina, Italy

**Keywords:** communication, team, audit, feedback, training, teamwork, organization

## Abstract

Communication constitutes an essential aspect of teamwork. This is especially true for audit teams, where communication takes place not only within the work group but also with the audit recipients. For this reason, given the poor evidence in the literature, communication training was carried out on an audit team. Training was divided into 10 meetings of two hours each, with the meetings taking place over 2 months. Questionnaires were administered to identify the characteristics and styles of communication, to assess the sense of perceived self-efficacy in general and at work, and to evaluate the knowledge inherent in the communication. This battery was administered before and after the training to evaluate its effectiveness and its effects on self-efficacy, communication style, and knowledge. Furthermore, a communication audit was performed on the feedback provided by the team, to highlight satisfaction, strengths, and any critical issues that emerged during the feedback phase. The results obtained suggest that training has an effect not only on individual knowledge but also on personological aspects. Communication among colleagues and general self-efficacy appear to be improved by the process. Self-efficacy also specifically improves in the work environment, with subjects feeling more able to manage relationships and collaborations with colleagues and supervisors. Moreover, the audit team members were satisfied with the training received, perceiving an improvement in their own communication skills during the feedback phases.

## 1. Audit, Feedback and Communication

The term Audit and Feedback (A&F) refers to a set of systematic interventions aimed at collecting data; these data are subsequently compared with reference standards and then returned to healthcare providers through feedback meetings [1]. In recent years, the Italian Ministry of Health funded the EASY-NET project, with the aim of evaluating the effectiveness of A&F strategies to improve clinical practice and equity in different settings [2]. Within the scope of the EASY-NET project, the parameters of interest involve both the patient and the care process. The relevant parameters, with regard to the care of patients, are the processes of care, clinical outcomes, safety, and effectiveness. Concerning medical performance, the relevant parameters include the equity, timeliness, and efficiency of medical performance [3]. Seven Italian regions are involved in the EASY-NET project: Lazio, Friuli Venezia Giulia, Piedmont, Emilia-Romagna, Lombardy, Calabria, and Sicily. Each of these regions corresponds to a work package (WP 1–7) that differentiates their areas of intervention within the EASY-NET project. The IRCCS Centro Neurolesi Bonino Pulejo was responsible for the Sicilian work package (WP7). The areas of intervention of WP7 are acute myocardial infarction (AMI) and ischemic stroke (IS) settings, both in acute and rehabilitation phases. The main objective of WP7 is to evaluate the previously mentioned parameters, in order to improve the quality of healthcare processes and services increasing safety as well as care access performance. Furthermore, WP7 was the promoter of a new model of A&F involving experienced psychologists and telemedicine protocols. The target population of the A&F intervention is healthcare workers, in the context of cardio- and cerebrovascular emergency (AMI and ischemic stroke) and in neurological rehabilitation (ischemic stroke).

To conduct the A&F procedures, generally a multidisciplinary team is formed, and the selection of team members is always given special attention. This occurs because each member needs to have the knowledge, competencies, and communication skills that make up a profile that is not always easily identified. Once the team is formed, the process can begin with the first phase: the audit. During this phase, the team defines the objectives, evaluates the existing documents by constructing an appropriate checklist based on the indicators to be produced, and establishes the strategy for data collection. Subsequently, the team conducts a document audit through a document analysis, processes the collected data, and produces a report containing the strengths and weaknesses [3]. In the second phase of the process (feedback), the results are shared and communicated with medical professionals, and an action plan is defined by guiding and supporting efforts toward change.

A&F constitutes a process in which communication plays a key role both within the audit team and toward the recipients of the process.

Initially, in the early 1900s, the communicative act was conceptualized as a passage of content from a sender to a receiver [4,5,6,7], but it is not limited to this. In fact, the phenomenon occurs when the expression is understood and becomes common heritage for the construction of a meaning, a discussion, or a culture, defining human relationships [8]. In 1967, the Austrian psychologist P. Watzlawick guessed that communication accounts for a mutually influential event and developed the systemic communication model [9]. The latter states that it is not impossible to isolate the subject from the context of relationships in which they are embedded, and, because of this, each participant influences and is influenced by the others. Following this thread through today, communication is understood as a complex systemic phenomenon that includes cognitive, emotional, and social resources. In 2013, NHS England introduced communication in the “6C for health professionals”, which are: Care, Compassion, Competence, Communication, Courage, and Commitment. These qualities help health professionals to improve the quality of patient care.

Communication among team members plays a key role in A&F, since being a skilled communicator helps facilitate information sharing [10], enables conflicts and misunderstandings to be avoided or resolved quickly [11], and is considered important for the continuity of care within multidisciplinary teams [12].

Working within a multidisciplinary team implies establishing necessary cooperative processes among a group of individuals with different backgrounds but common objectives [13]. Personal aspects always affect the teamwork process, generating misunderstandings and errors.

This is especially true in healthcare settings, where interpersonal and communication dynamics merge with time-dependent factors. Errors in both oral and written communication seem to be among the major causes of medication errors [14], even turning out to be the culprit in over half of all the causes associated with those errors [15] and an important modifiable factor in patient mortality [16]. For these reasons, it is important to increase knowledge about and raise awareness of the communication and interpersonal issues among the members of A&F teams as well as among the healthcare professionals working in the audited wards.

Communication also turns out to be important not only between colleagues but also for patients: ineffective communication led to less understanding of clinical status, a poor understanding of treatment needs, and lower patient satisfaction with hospitalization [17]. Furthermore, a recent study found that healthcare staff reported an inability to communicate in 49% of cases and a difficulty to communicate with patients in 35% of cases [18].

Given the extreme importance of communication in teamwork and in healthcare settings, the objective of our study was to evaluate the effects that a specific training procedure on improving communication and interpersonal/group aspects could have on a clinical audit team and to monitor the effects during the feedback process.

## 2. Materials and Methods

Within the EASY-NET project, WP7 was focused on the effectiveness of A&F strategies in both AMI and ischemic stroke settings, both in acute and rehabilitation phases. At this purpose, an audit team consisting of professionals working in different focus areas, with documented experience in audit, was formed. The audit team members’ professional categories were chosen according to the EASY-NET WP7 audit’s purpose. Physicians and nurses were chosen based on the departments covered by the audit (AMI, stroke, and rehabilitation), while statisticians, risk managers, and audit specialists were necessary for conducting the audit process. The study population consisted solely of the members of this team; thus, the sample size matches the number of audit team members. Specifically, the team was composed of 10 members: cardiologists (N = 2), neurologists (N = 2), nurses (N = 2), statisticians (N = 2), a clinical risk manager (N = 1), and an audit specialist (N = 1).

Once the audit team was formed, due to the strong relevance to the work environment, 4 questionnaires were administered to team members.

- Interpersonal Communication Inventory (ICI) [19] is a 54-item scale that measures the process of communication as an element of social interaction. This allows for identifying patterns, characteristics, and styles of communication. Moreover, this questionnaire does not consider communications and relationships with family and friends, which makes it more suitable for the assessment of communications between colleagues.
- General Self-Efficacy Scale (GSE) [20] is a 10-item scale that measures the general sense of perceived self-efficacy. Optimistic self-beliefs allow for coping with a variety of difficult demands at work and with colleagues. The total score is calculated by finding the sum of all items. Score ranges between 10 and 40, with a higher score indicating more self-efficacy.
- Scale of Perceived Self-Efficacy at Work [21] is a 10-item scale that evaluates perceived self-efficacy at work. Furthermore, the scale allows for obtaining two distinct scores for each subject: “relational readiness” and “commitment”. Relational readiness refers to the beliefs that people have about their ability to manage the relationships and collaborations within the work context with colleagues, bosses, and clients. It also refers to the willingness to work with people who have characteristics different from one’s own. By commitment, we refer to the beliefs that people have about their abilities to commit to and organize themselves in achieving work objectives by meeting set deadlines and concentrating on learning new work skills.
- A 12-item questionnaire was realized ad hoc to assess basic knowledge inherent in communication and dynamics affecting organizational settings.

The same team subsequently underwent a 2-month training course divided into 10 meetings of 2 h each.

After the administration of the 4 questionnaires, training materials were sent to each participant by e-mail, so they could familiarize themselves with the topics and issues to be addressed later during the training phase. Topics covered were:

o Communication and its elements;
o Verbal, paraverbal, and nonverbal communication;
o Listening and its modalities;
o Communication styles;
o Grice’s conversational maxims;
o The dysfunctional modes of communication;
o The group and its dynamics (requirements, episodes, and defenses);
o Effective communication.

The training phase consisted of 4 face-to-face lectures, 3 focus group, and 3 workshops. During lectures, three psychologists who specialized in communication explained the topics covered in the material that was previously sent to team members. During focus group meetings, participants were invited to speak about and discuss their personal attitude toward communication and interpersonal dynamics in presence of a supervisor. In the workshops phase, role-playing activities were proposed in which an attempt was made to reproduce communication problems and happenings similar to those in real life. After the training procedure was completed, the 12-item questionnaire, ICI, GSE, and Scale of Perceived Self-Efficacy at Work were re-administered to the audit team members, to assess whether the training had contributed to the improvement of communication and interpersonal/group aspects.

Furthermore, since this was deemed to be in line with the objectives of the study, a communication audit was performed on feedback provided by the team, following guidelines proposed by Hargie and Tourish [22].

**Figure 1:**
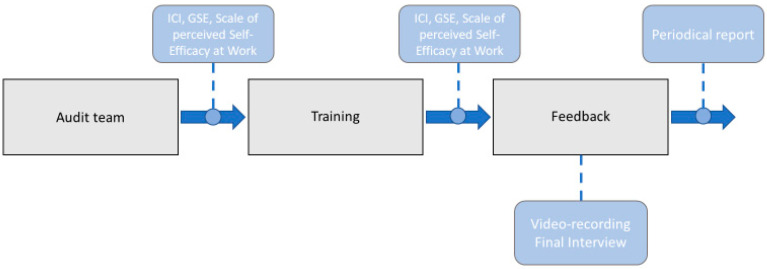
Structure of the study.

Figure 1 shows the structure of the study.

The EASY-NET network research project provided for half of the hospitals sampled to have feedback that would be given both in written form via email and in oral form via telemedicine. All six online feedback sessions were video-recorded and supervised by the same psychologists who did the training. Immediately after the feedback ended, the team was given a brief interview during which they were asked about their satisfaction, the strengths, and any critical issues that emerged during the feedback. At the end of the interview, psychologists would give their views on the feedback and, where necessary, offer suggestions and insights. Data registered by interviews and videorecording were drafted, summarized, and periodically given as feedback to the team members.

**Figure 2:**
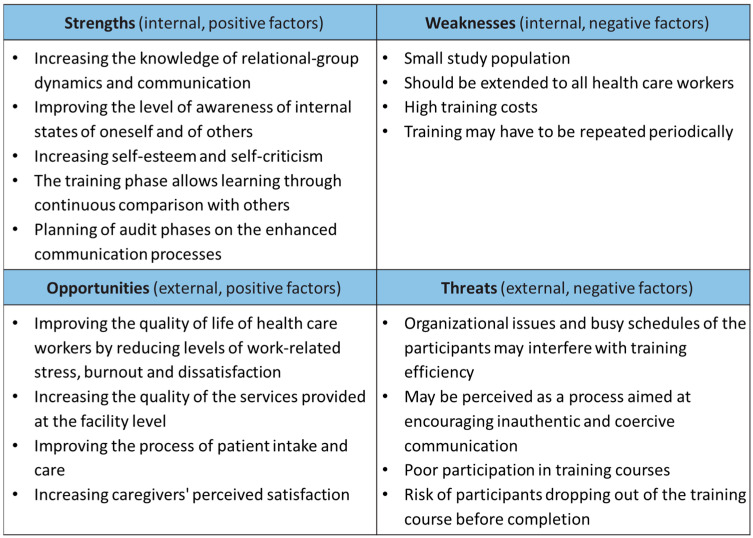
SWOT analysis chart.

A SWOT analysis was conducted to evaluate the strengths and weaknesses of the study and to assess what opportunities and threats may be encountered during the process. Figure 2 shows the results of the SWOT analysis.

### Statistical Analysis

Data were analyzed using the R software—version 4.2.2, considering *p*-value < 0.05 as statistically significant. The Wilcoxon signed-rank test was used to compare scores between baseline and follow-up.

## 3. Results

All the audit team members (mean ± SD age: 51.4 ± 2.8 years; 40.0% male) responded to the questionnaires. Table 1 shows the demographic characteristics of the studied population.

**Figure 3:**
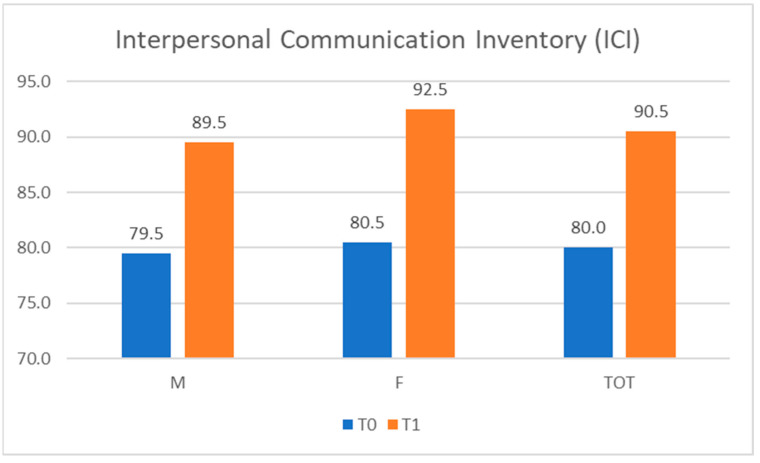
Interpersonal Communication Inventory (ICI) scores. M = male; F = female; TOT = total.

The ICI test at T0 resulted in a score between 75 and 87, with a median value of 80.1. As shown in Figure 3, the ICI median scores obtained by males and females at T0 were 79.5 and 80.5, respectively. The ICI score at T1 was in the range of 83 to 95, with a median value of 90.5 (89.5 for males and 92.5 for females).

**Figure 4:**
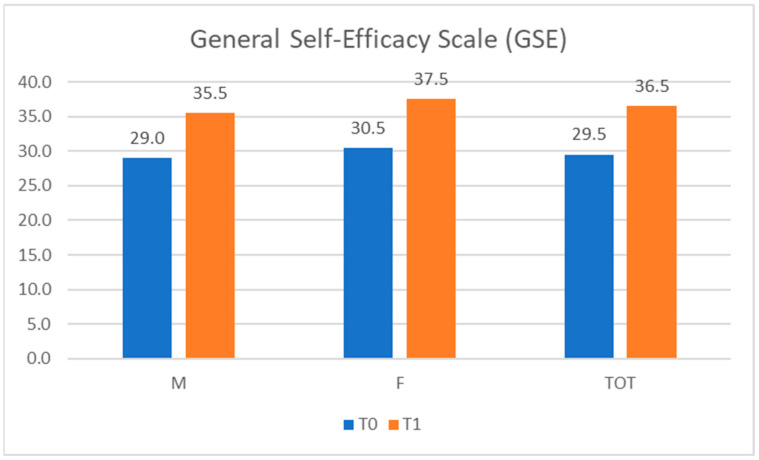
General Self-Efficacy Scale (GSE) scores. M = male; F = female; TOT = total.

GSE test scores covered a range from 27 to 32, with a median value of 29.5 (29.0 for males and 30.5 for females) at T0, and a range from 34 to 38, with a median value of 36.5 (35.5 for males and 37.5 for females) at T1, as shown in Figure 4.

**Figure 5:**
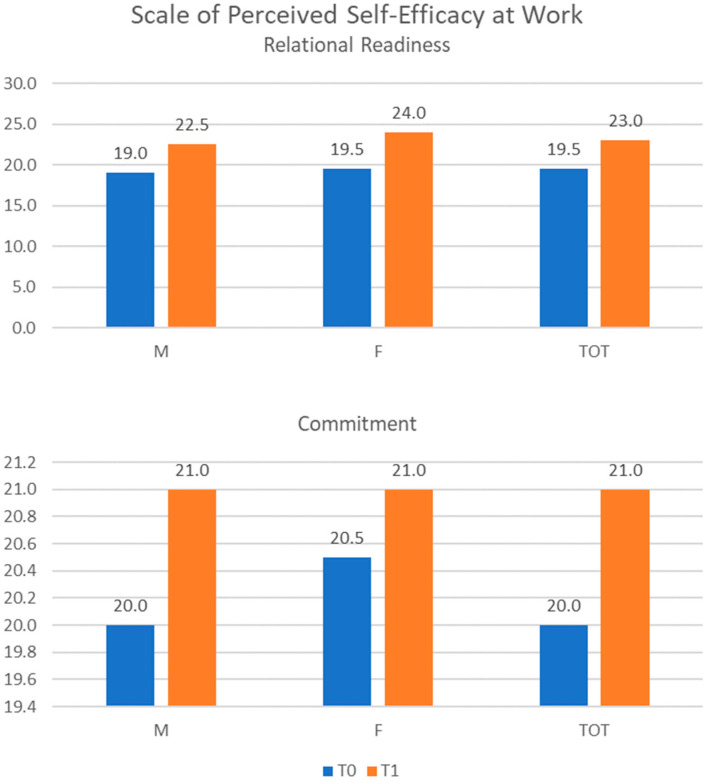
Scale of Perceived Self-Efficacy at Work scores. M = male; F = female; TOT = total.

As mentioned, the perceived work self-efficacy scale used in the study allows for assessing two dimensions: relational readiness and commitment. At T0 relational readiness, the scores ranged from 16 to 21, with a median value of 19.5 (19.0 for males and 19.5 for females); at T1 relational readiness, the scores ranged from 21 to 25, with a median value of 23.0 (22.5 for males and 24.0 for females). Commitment scores ranged from 18 to 22, with a median value of 20.0 (20.0 for males and 20.5 for females), at T0 and from 20 to 23, with a median value of 21.0 (21.0 for males and 21.0 for females), at T1 (Figure 5).

**Figure 6:**
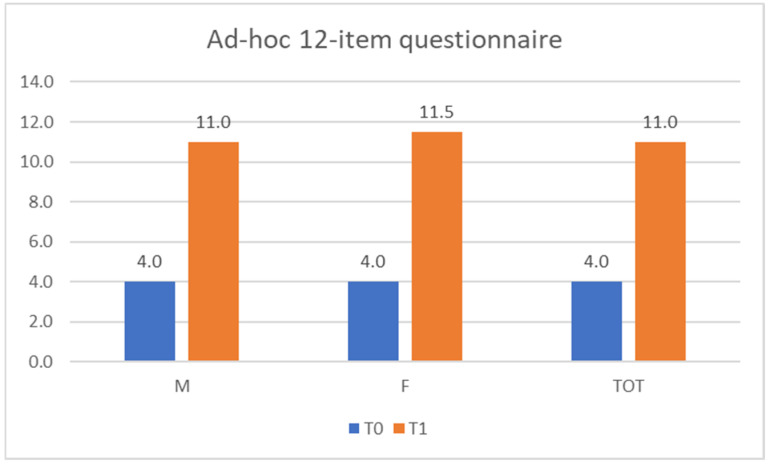
Ad hoc 12-item questionnaire scores. M = male; F = female; TOT = total.

At T0, the 12-item questionnaire was aimed at assessing basic knowledge, which resulted in scores ranging from 3 to 7, with a median value of 4.0 (4.0 for males and 4.0 for females). At T1, the effectiveness of the training interventions was evaluated by performing the same 12-item questionnaire. As shown in Figure 6, the scores ranged from 10 to 12, with a median value of 11.0 (11.0 for males and 11.50 for females).

**Figure 7:**
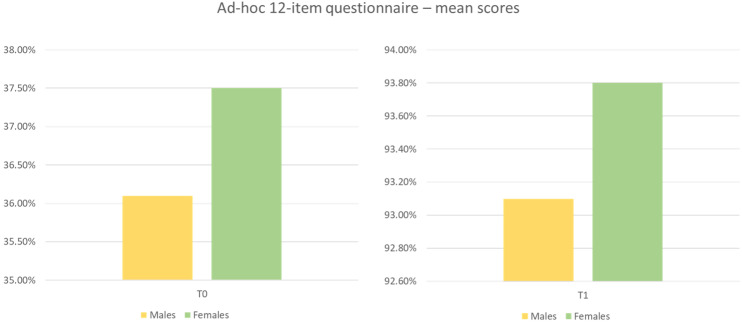
Comparison of average percentage scores obtained by males and females on the 12-item questionnaire.

Compared with the initial data, there was an average improvement of 56.7% in the performance of the subjects (Figure 7).

As shown in Table 2, the statistical analysis revealed significant differences between T0 and T1 for each of the questionnaires administered.

The effectiveness of the treatment was further evaluated by observing the percentage improvement achieved on each questionnaire’s scores. The scores obtained by the ICI improved by 12.6% (11.7% in males and 13.9% in females), while those obtained by the GSE improved by 23.4% (23.6% in males and 23.1% in females). Regarding the perceived work self-efficacy scale, the relational readiness score improved by 21.1% (20.5% in males and 21.8% in females) and the commitment score improved by 5.5% (6.8% in males and 3.7% in females).

The short interviews that were performed at the end of the feedback enabled qualitative assessment of the satisfaction, strengths and critical issues that emerged during the feedback sessions. The satisfaction with the communication that was carried out gradually increased, and all participants found the training they received to be useful within their relationships with the feedback recipients. Among the critical issues reported during the remote feedback sessions, some technical difficulties emerged from the audit recipients, on both the hardware/software side (driver problems or poor Internet access) and the user side (lack of knowledge about using the software and perceived poor cooperation).

## 4. Discussion and Conclusions

Although the importance of communication and communication training for teams and teamwork has been widely documented and demonstrated in the literature [23,24,25,26,27], the impact of training on an audit team of professionals has never been specifically addressed.

The results obtained in this study seem to suggest that communication training for the team has an effect not only on individual knowledge but also on personological aspects. In line with the findings in the literature, communication with colleagues and general self-efficacy appear to be improved by the process, as evidenced by the scores obtained by the ICI and GSE. Self-efficacy also specifically improves in the work environment; indeed, the scores obtained by the Scale of Perceived Self-Efficacy at Work show that subjects feel more able to manage the relationships and collaborations with colleagues and supervisors. There was less improvement in commitment, probably because it was not in line with the training that was carried out.

Improvements were also recorded in commitment, although to a lesser extent, probably because it was not in line with the training that was performed. The results obtained by the 12-item questionnaire demonstrated an increase in knowledge about communication and organizational dynamics. In addition, the results show that women’s performance was slightly better both at baseline and after the training. Bertrand et al. [28] showed that better communication skills are associated with better team performance. People have begun to be aware of this, which is why more and more innovative methods are being sought out to carry out these trainings, such as simulators [29] and artificial intelligence [30]. Although it is universally recognized that communication and training are key aspects within work settings, little time and money continue to be invested on it. The limitations of training are its costs, which are often large, and especially the need for it to be renewed over time. However, the greater quality and generalizability of the effects of training could be the key to increasing the sensitivity of intervening in this manner.

Due to this, the present paper aims to assess whether and how much a structured training program affects communication and relational dynamics.

The innovation of this study lies in the fact that audit and feedback courses, as well as medical training courses, hardly take into account data on the communicative, relational, and personal aspects that influence the performance of healthcare workers. Indeed, audit team members, despite being highly specialized professionals, did not display high levels of communication knowledge during the first assessment. Moreover, the inclusion of a psychologist in the audit procedures constitutes a pioneering aspect of this study.

As an implication, this study is intended to stimulate reflection on the global importance that the inclusion of a training procedure regarding these dynamics can have on audit teams, healthcare workers, and clinical performance quality. These are preliminary data; indeed, the size of the sample taken is a limitation of this study, so this study could lay the groundwork for future studies. It would be interesting to evaluate the effectiveness of the training on a larger sample consisting of different teams, so their performances and improvements can be compared. The effects of training after a period of time could also be evaluated, to see if training improvements are actually introduced into work practice and in teamwork in the long run.

## Figures and Tables

**Table 1:** Demographic characteristics. Mean ± standard deviation was used to describe continuous variables; proportions (numbers and percentages) were used to describe categorical variables.

	Cardiologist	Neurologist	Nurse	Statistician	Risk Manager	Audit Specialist	All
Subjects	2	2	2	2	1	1	10
Age	52.5 ± 3.5	53 ± 1.4	51 ± 2.8	47.5 ± 0.7	54	52	51.4 ± 2.8
Gender							
Male	1 (50.0)	1 (50.0)	2 (100.0)	1 (50.0)	1 (100.0)		6 (60.0)
Female	1 (50.0)	1 (50.0)	NA	1 (50.0)		1 (100.0)	4 (40.0)

**Table 2:** Statistical comparisons of clinical scores between baseline (T0) and follow-up (T1). Scores are in median (first–third quartiles); Significant differences between treatment effects are in bold. ICI = Interpersonal Communication Inventory; GSE = General Self-Efficacy Scale; SEW-RR = Scale of Perceived Self-Efficacy at Work—relational readiness; SEW-CO = Scale of Perceived Self-Efficacy at Work—commitment; Q12 = ad hoc 12-item questionnaire.

Questionnaire	T0	T1	*p*-Value
ICI	80.0 (78.3–80.8)	90.5 (89.0–92.0)	0.006
GSE	29.5 (28.3–30.8)	36.5 (35.3–37.8)	0.005
SE-RR	19.5 (18.0–20.0)	23.0 (22.0–23.8)	0.005
SEW-CO	20.0 (19.3–20.8)	21.0 (20.3–21.0)	0.001
Q12	4.0 (3.3–5.0)	11.0 (11.0–12.0)	0.006

## Data Availability

Data available in a publicly accessible repository. The data presented in this study are openly available in Zenodo, at https://doi.org/10.5281/zenodo.7673801.
